# Supplementary material for: Tudor staphylococcal nuclease is a docking platform for stress granule components and is essential for SnRK1 activation in Arabidopsis
Source: EMBO J. 2021 Jul 21;40(17):e105043. doi: 10.15252/embj.2020105043 (PMC8447601; doi:10.15252/embj.2020105043)
Supplement: Supplementary file 2 — Appendix [file EMBJ-40-e105043-s001.pdf]

Appendix: Tudor staphylococcal nuclease is a docking platform for stress granule components and is essential for SnRK1 activation in *Arabidopsis*

Emilio Gutierrez-Beltran, Pernilla H. Elander, Kerstin Dalman, Guy W. Dayhoff II, Panagiotis N. Moschou, Vladimir N. Uversky, Jose Luis Crespo and Peter V. Bozhkov

Table of Contents

Figure S1. qPCR analysis of HS-induced genes *HSP101* and *HSF*.

Figure S2. Protein-protein interaction networks within TSN2_NS and TSN2_HS protein pools isolated by TAPa.

Figure S3. *Arabidopsis* lines used for isolation of RBP47 interactome.

Figure S4. Co-localization of GFP-TSN2-interacting proteins (green) and RFP-TSN2 (red) quantified in Fig. 3C.

Figure S5. BiFC analysis of TSN2 and TSN2-interacting proteins under no stress conditions.

Figure S6. Disorder in the *Arabidopsis* TSN proteins.

Figure S7. Co-localization of RH12 and RBP47 in *Arabidopsis* root cells.

Figure S8. Activation of *Arabidopsis* SnRK1a isoforms under ABA treatment.

Table S1. Primers used in this study.

Table S2. Vectors and constructs used in this study.

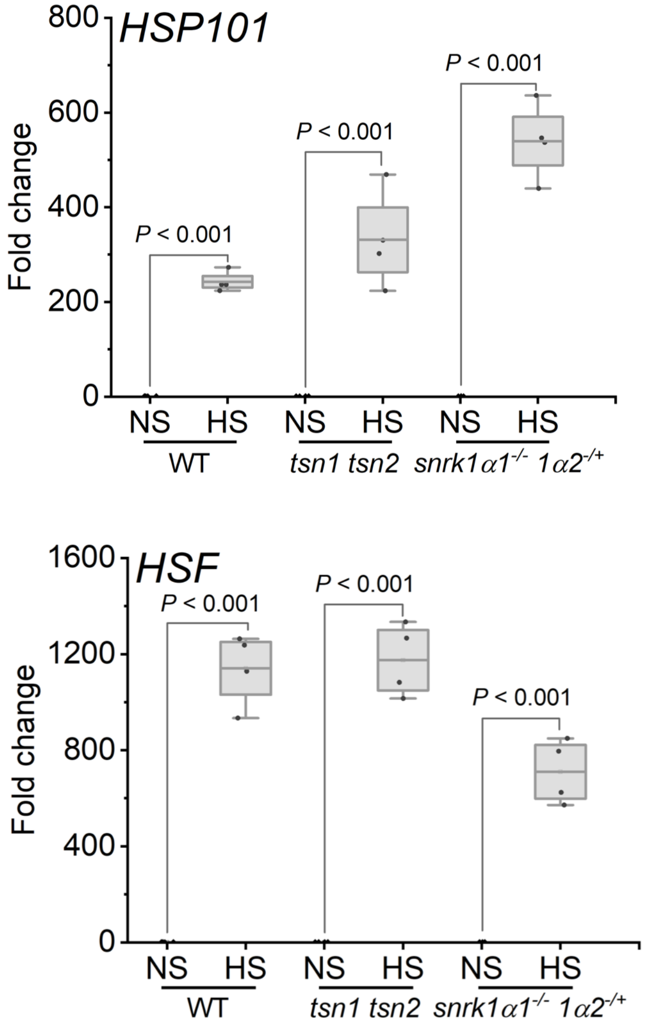

Appendix Figure S1. qPCR analysis of HS-induced genes *HSP101* and *HSF*.

Gene expression was measured in 10-day-old *Arabidopsis* Col, *tsn1 tsn2* or *snrk1α1*$^{-/-}$ *1α2*$^{-/+}$ seedlings subjected to HS (39ºC for 60 min) or grown under control (23ºC, NS) conditions. Upper and lower box boundaries represent the first and third quantiles, respectively. Horizontal lines mark the median and whiskers mark the highest and lowest values. *P* values denote statistically significant differences for comparisons to NS (two-tailed *t*-test). Three independent experiments showed similar results.

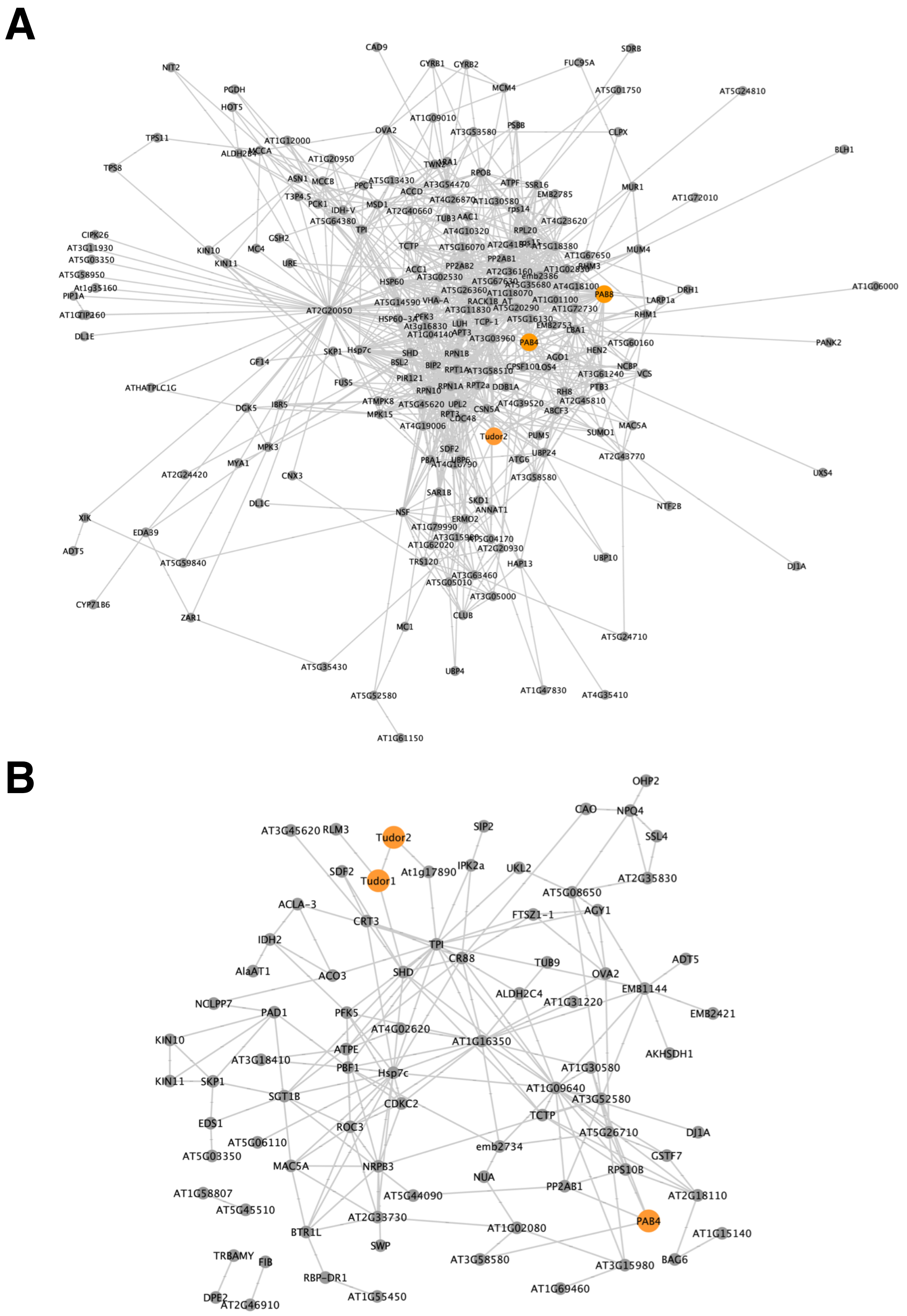

Appendix Figure S2. Protein-protein interaction networks within TSN2_NS (A) and TSN2_HS (B) protein pools isolated by TAPa. Proteins previously known to localize in plant SGs are marked by orange circles. Tudor1 and Tudor2 are TSN1 and TSN2, respectively. The networks were visualized in Cytoscape using a force-directed layout.

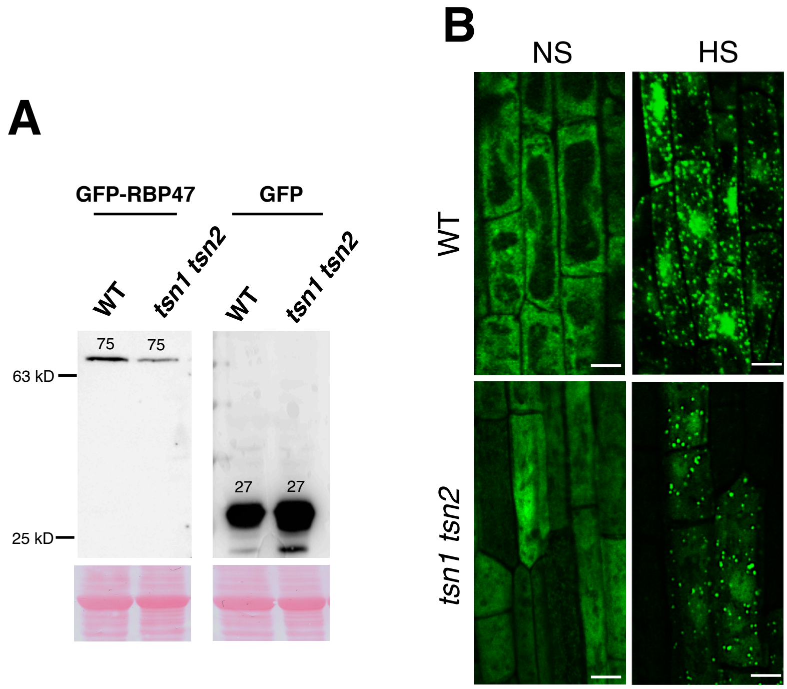

Appendix Figure S3. *Arabidopsis* lines used for isolation of RBP47 interactome.

A, Expression of GFP-RBP47 and GFP in WT and *tsn1 tsn2* backgrounds confirmed by immunoblotting with α-GFP. Each lane was loaded with 20 µg total protein from a crude homogenate. The expected molecular weights (kD) of the expressed proteins are indicated on the blots. Ponceau S staining was used as a loading control (bottom panel).

B, Localization of GFP-RBP47 in root cells of 5-day-old *Arabidopsis* WT and *tsn1 tsn2* seedlings. The seedlings were grown under 23ºC (NS) or incubated at 39ºC for 60 min (HS). Scale bars = 10 µm.

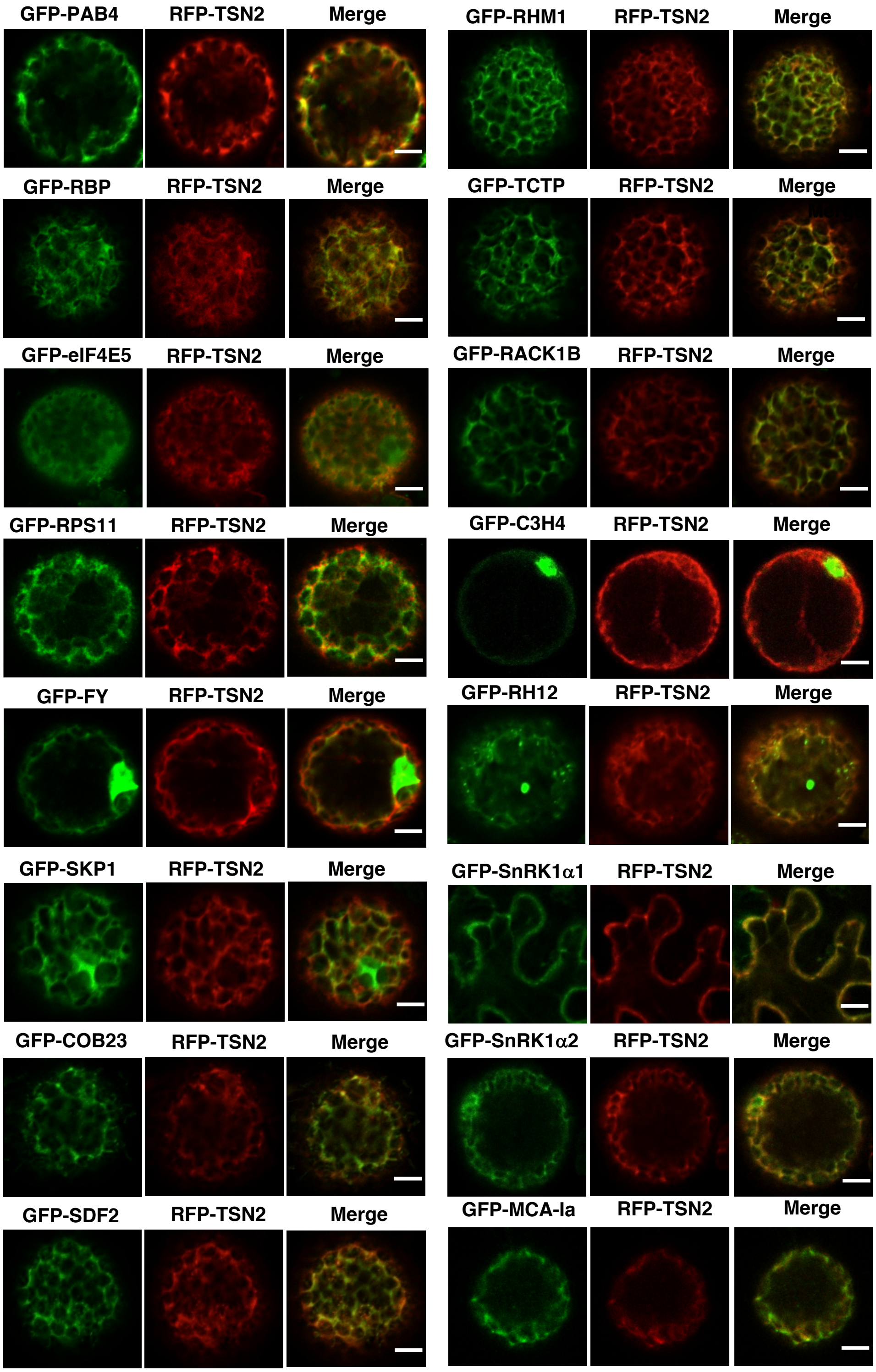

Appendix Figure S4. Co-localization of GFP-TSN2-interacting proteins (green) and RFP-TSN2 (red) quantified in Fig. 3C.

Co-localization analysis in *N. benthamiana* leaf protoplasts or epidermal cells under no stress conditions (23°C). Scale bars = 5 µm.

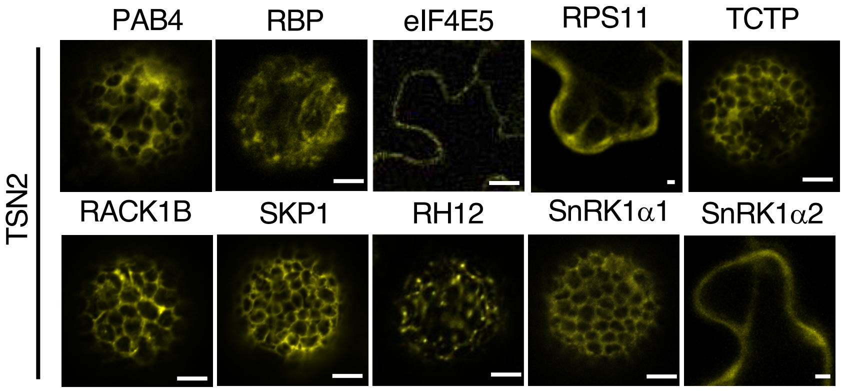

Appendix Figure S5. BiFC analysis of TSN2 and TSN2-interacting proteins under no stress conditions. BiFC between cYFP-TSN2 and individual nYFP-TSN-interacting proteins in *N. benthamiana* leaf protoplasts or epidermal cells incubated at 23°C. Scale bars = 5 µm.

A

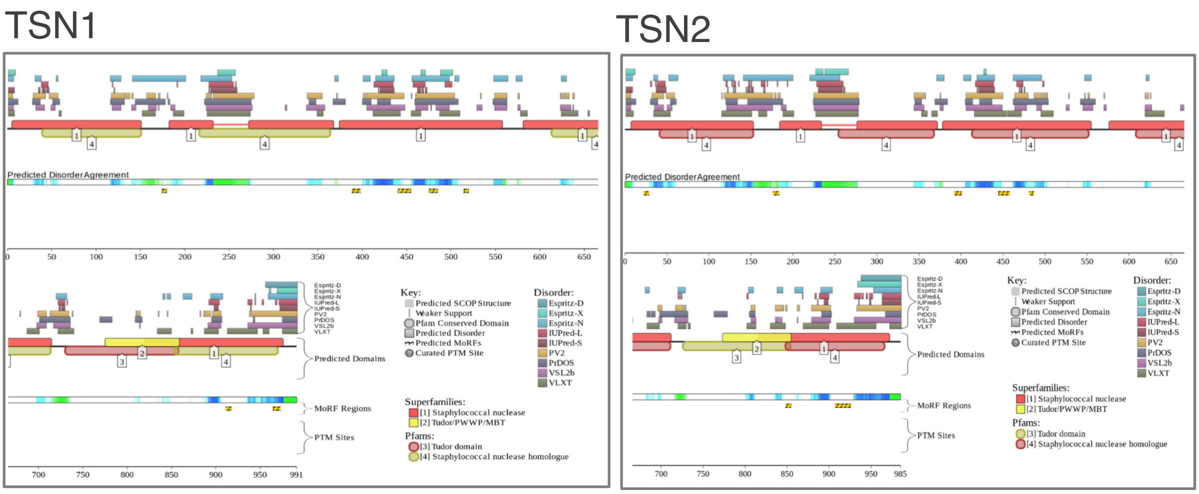

B

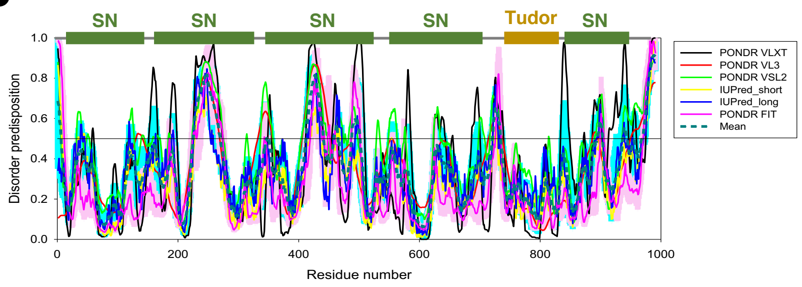

Appendix Figure S6. Disorder in the *Arabidopsis* TSN proteins.

A, Evaluation of the functional ID propensity of TSN1 and TSN2 using the D2P2 database. In the corresponding plots, top nine colored bars represent the location of IDRs predicted by different disorder predictors (Espritz-D, Espritz-N, Espritz-X, IUPred-L, IUPred-S, PV2, PrDOS, PONDRs VSL2b, and PONDRs-VLXT); see keys for the corresponding color codes. Green/blue-and-white bar in the middle of each plot shows the predicted disorder agreement between these nine predictors, with green/blue parts corresponding to IDRs by consensus. The yellow bar shows the location of the predicted disorder-based binding site (MoRF region).

B, Evaluation of the per-residue disorder propensity of TSN1 using six different disorder predictors, and a consensus disorder profile (based on mean values of six predictors). SN, staphylococcal nuclease domain.

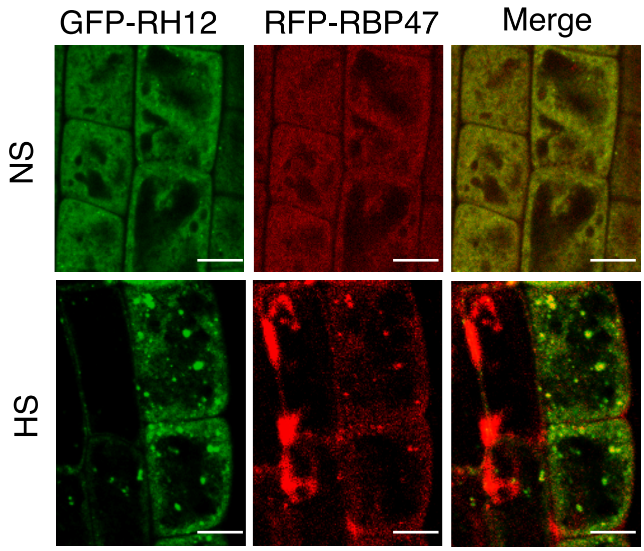

Appendix Figure S7. Co-localization of RH12 and RBP47 in *Arabidopsis* root cells. 5-day-old seedlings co-expressing *ProRH12:RH12-GFP* and *Pro35S:RFP-RBP47* were grown under control conditions (23ºC, NS) or subjected to HS (39ºC for 60 min). Scale bars = 10 µm.

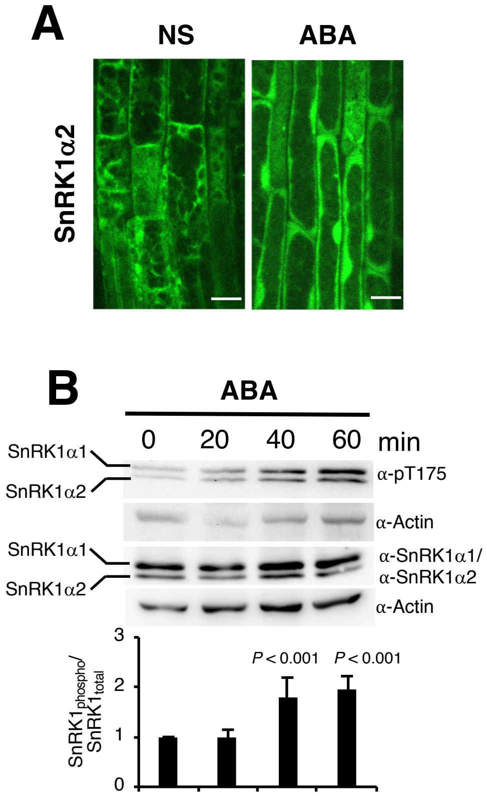

Appendix Figure S8. Activation of *Arabidopsis* SnRK1α isoforms under ABA treatment.

A, Localization of SnRK1α2 in the root cells of 5-day-old WT seedlings expressing *ProUBQ:GFP-SnRK1α2*. The seedlings were grown under no stress (NS) conditions or incubated for 40 min with 10 µM ABA (ABA). Scale bars = 10 µm.

B, Immunoblot analysis with indicated antibodies of protein extracts prepared from 10-day-old *Arabidopsis* WT seedlings treated with 10 µM ABA for 0, 20, 40 and 60 min. The chart shows SnRK1 activity, expressed as the ratio of phosphorylated to total SnRK1 protein. The data represent mean ratios of integrated band intensities (for both isoforms) normalized to 0 min ± SD from three experiments. *P* values denote statistically significant differences for comparisons to 0 min (two-tailed *t*-test).

Appendix Table S1. Primers used in this study.

Name	Sequence
Primers for TAP constructs	
TSN1 -F	5´-GGGGACCACTTTGTACAAGAAAGCTGGGTATTACCGGCGACCACCAGCA-3´
TSN1 -R	5´-GGGGACCACTTTGTACAAGAAAGCTGGGTACCGGCGACCACCAGCAGGC-3´
TSN2 -F	5´-GGGGACAAGTTTGTACAAAAAAGCAGGCTTGATGGCGACTGGGGCAGCA-3´
TSN2 -R	5´-GGGGACCACTTTGTACAAGAAAGCTGGGTACCCGCGACCCGGTTTC-3´
GFP -F	5´-GGGGACAAGTTTGTACAAAAAAGCAGGCTTGatggtgagcaagggcgag-3´
GFP -R	5´-GGGGACCACTTTGTACAAGAAAGCTGGGTActtgtacagctcgtccat-3´

Name	Sequence
Primers for BiFC constructs	
TSN2 -F	5´-GGGGACAAGTTTGTACAAAAAAGCAGGCTTGATGGCGACTGGGGCAGCA-3´
TSN2 -R	5´-GGGGACCACTTTGTACAAGAAAGCTGGGTATTACCCGCGACCCGGTTTC-3´
SN2 -F	5´-GGGGACAAGTTTGTACAAAAAAGCAGGCTTGATGGCGACTGGGGCAGCA-3´
SN2 -R	5´-GGGGACCACTTTGTACAAGAAAGCTGGGTATTAGTTTACTACTTCCTCTCCTTCA-3´
Tudor -F	5´-GGGGACAAGTTTGTACAAAAAAGCAGGCTTGATGGGTAGTTCTAAAGTAGAAACCAGGCA-3´
Tudor -R	5´-GGGGACCACTTTGTACAAGAAAGCTGGGTATTACCCGCGACCCGGTTTC-3´
TCTP -F	5´-TCAATAAGCGACCATGTTGGTGTACCAAGATCTTCTCACCGGT-3´
TCTP -R	5´-GGGGACCACTTTGTACAAGAAAGCTGGGTAGCACTTGACCTCCTTCAAAC-3´
RH12 -F	5´-AGAGAGATTTGTGATGAATACTAACAGAGGAAGATATCCACCG-3´
RH12 -R	5´-GGGGACCACTTTGTACAAGAAAGCTGGGTACTGACAGTAGATTGCTTGA-3´
SnRK1α2 -F	5´-TGGGACACAGGAAATGGATCATTCATCAAATAGATTTGGCAAT-3´
SnRK1α2 -R	5´-GGGGACCACTTTGTACAAGAAAGCTGGGTAGATCACACGAAGCTCTGTA -3´

Name	Sequence
Primers for Co-IP constructs	
TSN1-Myc -F	5´-GGGGACCACTTTGTACAAGAAAGCTGGGTATTACCGGCGACCACCAGCA-3´
TSN1-Myc -R	5´-GGGGACCACTTTGTACAAGAAAGCTGGGTACCGGCGACCACCAGCAGGC-3´
TSN2-Myc -F	5´-GGGGACAAGTTTGTACAAAAAAGCAGGCTTGATGGCGACTGGGGCAGCA-3´
TSN2-Myc -R	5´-GGGGACCACTTTGTACAAGAAAGCTGGGTATTACCCGCGACCCGGTTTC-3´
RH12-GFP -F	5´-AGAGAGATTTGTGATGAATACTAACAGAGGAAGATATCCACCG-3´
RH12-GFP -R	5´-GGGGACCACTTTGTACAAGAAAGCTGGGTACTGACAGTAGATTGCTTGA-3´

Name	Sequence
Primers for plant transformation constructs	
ProRH12 -F	5´-GGGGACAAGTTTGTACAAAAAAGCAGGCTTGGGATTCATGTTCTTCAGCT-3´
ProRH12 -R	5´-GGGGACCACTTTGTACAAGAAAGCTGGGTAGTATTCATCACAAATCTCTCTC-3´
RH12 -F	5´-AGAGAGATTTGTGATGAATACTAACAGAGGAAGATATCCACCG-3´
RH12 -R	5´-GGGGACCACTTTGTACAAGAAAGCTGGGTACTGACAGTAGATTGCTTGA-3´
TCTP -F	5´-TCAATAAGCGACCATGTTGGTGTACCAAGATCTTCTCACCGGT-3´
TCTP -R	5´-GGGGACCACTTTGTACAAGAAAGCTGGGTAGCACTTGACCTCCTTCAAAC-3´
SnRK1α2 -F	5´-TGGGACACAGGAAATGGATCATTCATCAAATAGATTTGGCAAT-3´
SnRK1α2 -R	5´-GGGGACCACTTTGTACAAGAAAGCTGGGTAGATCACACGAAGCTCTGTA -3´
SnRK1α1 -F	5´-AAAAAGCAGGCTTGATGGATGGATCAGGCACA -3´
SnRK1α1 -R	5´-AGAAAGCTGGGTATCAGAGGACTCGGAGCTGAG -3´

Name	Sequence
Primers for constructs used in co-localization experiment	
SnRK1α1^{CD} -F	5´-AAAAAGCAGGCTTGATGGATGGATCAGGCACA -3´
SnRK1α1^{CD} -R	5´-AGAAAGCTGGGTATGCCTGTTGCACAGTAT -3´
SnRK1α1RD -F	5´-AAAAAGCAGGCTTGATGAAAAAGATTGACGAGGA -3´
SnRK1α1RD -R	5´-AGAAAGCTGGGTAGAGGACTCGGAGCTGAGCAAG -3´

Name	Sequence
Primers for qPCR	
DIN2 qPCR-F	5´-TGAAGGTGGCAAGTCTCCAA -3´
DIN2 qPCR-R	5´-AGCGTCCATGTTTAGCTCCT -3´
DIN6 qPCR-F	5´-AACTTGTCGCAAGATCAAGG -3´
DIN6 qPCR-R	5´-GGAACACGTGCCTCTAGTCC -3´
UBQ10 qPCR-F	5´-AACTTTGGTGGTTTGTGTTTTGG -3´
UBQ10 qPCR-R	5´-TCGACTTGTCATTAGAAAGAAAGAGATAA -3´

Appendix Table S2. Vectors and constructs used in this study.

Vectors and constructs	Experiments
pC-TAPa	Tandem affinity purification
pMDC43 (N-GFP)	Co-localization, Co-Ip and expression of GFP -tagged proteins
pGWB655 (N-RFP)	Co-localization
pSITE-cYFP, pSITE-nYFP	BiFC
pGWB4	Expression of GFP -tagged proteins under native promoter
pGWB18 (N-Myc)	Co-Ip
pGWB642 (N-YFP)	FRET
pGWB645 (N-CFP)	FRET
pUBC-GFP-Dest (N-GFP)	Co-localization, Co-Ip, FRAP and expression of GFP -tagged proteins
**Vectors and constructs**	**Experiments**
Pro35S:TSN1-TAPa<br>Pro35S:TSN2-TAPa<br>Pro35S:GFP-TAPa	Tandem affinity purification
Pro35S:GFP-RBP47	Isolation of RBP47 interactome
Pro35S:RFP-TSN2<br>Pro35S:GFP-TSN-interacting proteins	Co-localization
Pro35S:cYFP-TSN2<br>Pro35S:cYFP-SN<br>Pro35S:cYFP-Tudor<br>Pro35S:nYFP-TSN-interacting proteins	BiFC
ProRH12:RH12-GFP<br>Pro35S:GFP-RBP47<br>Pro35S:GFP-UBP1<br>Pro35S:GFP-TCTP<br>ProRBP:GFP-RBP	Expression in plants and Co-IP
ProUBP:GFP-SnRK1α1<br>ProUBP:GFP-SnRK1α1^{CD}<br>ProUBP:GFP-SnRK1α1RD<br>ProUBP:GFP-SnRK1α2	Expression in plants, Co-IP and FRAP
Pro35S:CFP-TSN2<br>Pro35S:YFP-SnRK1α1<br>Pro35S:YFP-SnRK1α2<br>Pro35S:YFP-TCTP<br>Pro35S:YFP-RBP	FRET
