# Supplementary material for: Tudor staphylococcal nuclease is a docking platform for stress granule components and is essential for SnRK1 activation in Arabidopsis
Source: EMBO J. 2021 Jul 21;40(17):e105043. doi: 10.15252/embj.2020105043 (PMC8447601; doi:10.15252/embj.2020105043)
Supplement: Supplementary file 3 — Expanded View Figures PDF [file EMBJ-40-e105043-s003.pdf]

Expanded View Figures

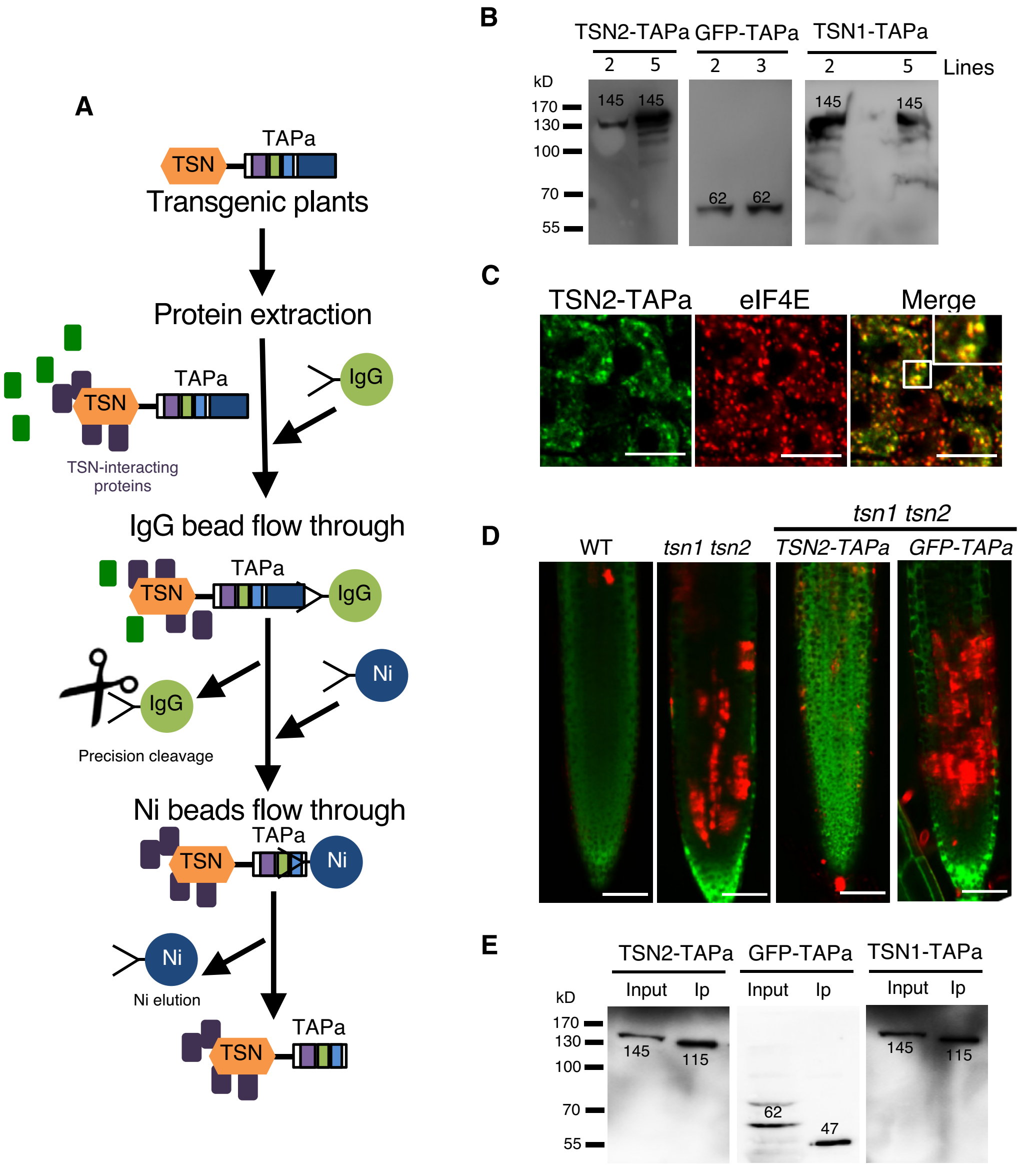

Figure EV1. TAPa procedure and *Arabidopsis* TSN-TAPa lines.

A Schematic representation of the TAPa procedure. During the first affinity purification step, plant protein extracts are incubated with IgG beads followed by elution through the specific cleavage of TAPa tag with the low-temperature active rhinovirus 3C protease. At the second affinity purification step, IgG bead eluates are incubated with Ni beads followed by the elution of proteins from beads using an imidazole-containing buffer.

B Expression of TSN-TAPa or GFP-TAPa in 10-day-old *Arabidopsis* seedling lines was confirmed by immunoblotting with α-Myc. Each lane was loaded with 20 µg total protein from a crude homogenate. The expected molecular weights (kD) of the expressed proteins are indicated on the blots.

C Immuno co-localization analysis of TSN2-TAPa (green, α-Myc) and eIF4E (red, α-eIF4E) in heat-stressed (39°C for 60 min) root tip cells of 5-day-old *Arabidopsis* seedlings expressing *Pro35S:TSN2-TAPa*. Scale bars = 10 µm.

D Five-day-old *Arabidopsis* seedlings expressing *Pro35S:TSN2-TAPa* or *Pro35S:GFP-TAPa* in the *tsn1 tsn2* background were exposed to a long-term HS treatment (39°C for 4 h), and roots were stained with both FDA (green; indicative of living cells) and SYTOX Orange (red; indicative of dead cells). WT and *tsn1 tsn2* were used as controls. The experiment was repeated three times with similar results. Scale bars = 50 µm.

E Immunoblotting of crude protein extracts (Input) and purified protein fractions (Ip) obtained during small-scale TAPa procedure from 10-day-old *Arabidopsis* seedlings expressing TSN-TAPa or GFP-TAPa. The numbers on the blots indicate expected molecular weights (kD) of the proteins detected using α-Myc.

Source data are available online for this figure.

 © 2021 The Authors

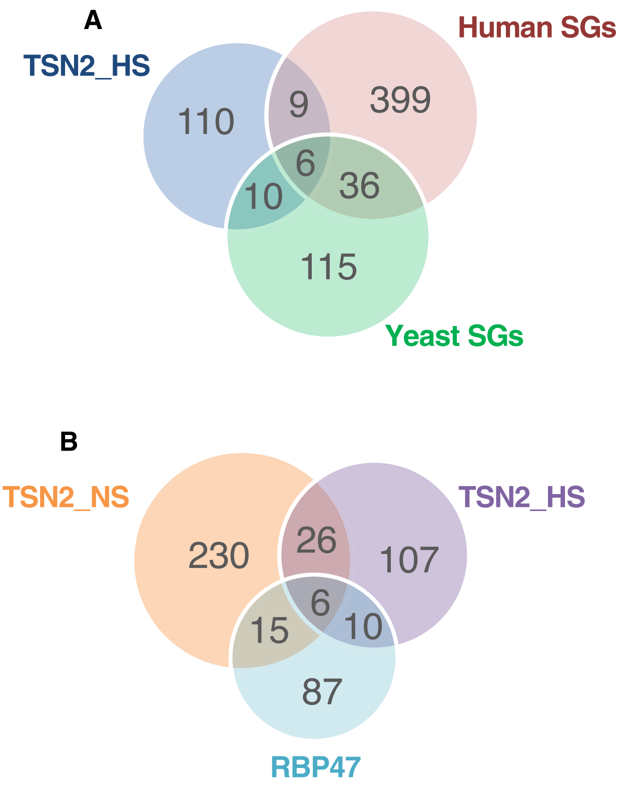

Figure EV2. Venn diagrams.

A Comparison between TSN2_HS interactome and both human and yeast SG proteomes (Jain *et al*, 2016).

B Comparison between TSN2_NS and TSN2_HS interactomes and *Arabidopsis* RBP47-SG proteome (Kosmacz *et al*, 2019).

© 2021 The Authors

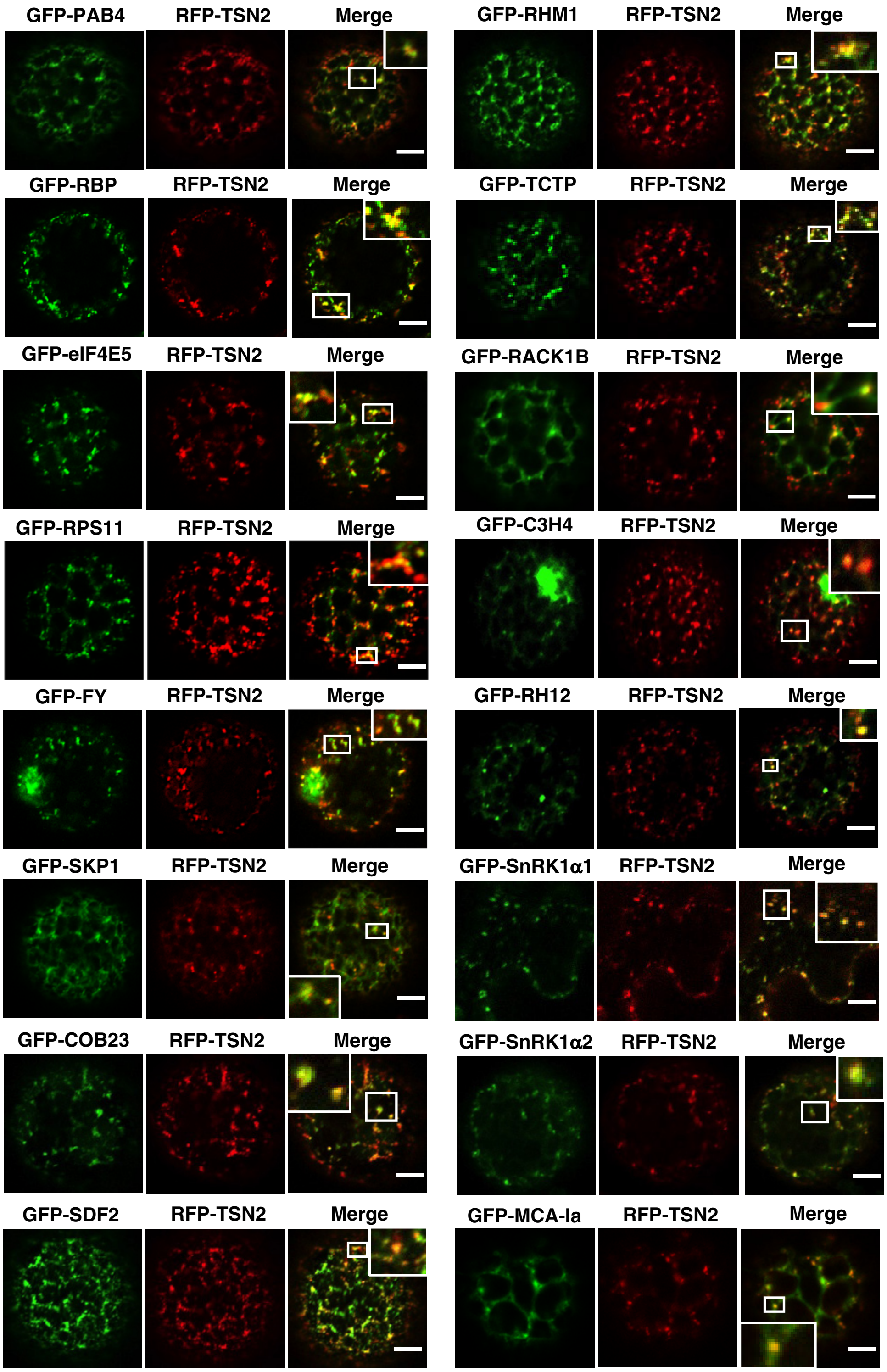

Figure EV3. Co-localization of GFP-TSN2-interacting proteins (green) and RFP-TSN2 (red) quantified in Fig 3C.

Co-localization analysis in *N. benthamiana* leaf protoplasts or epidermal cells under heat stress (39°C for 40 min). Insets show enlarged boxed areas. Scale bars = 5 μm.

 © 2021 The Authors

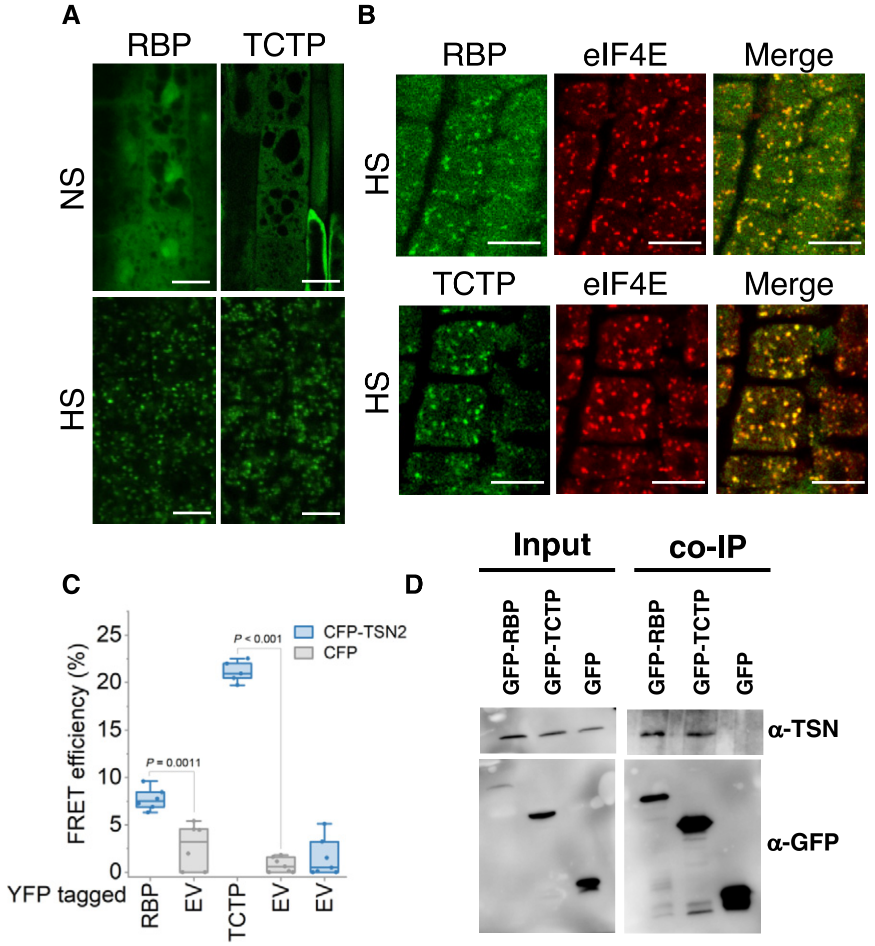

Figure EV4. RBP and TCTP, two novel plant SG components, interact with TSN *in vivo*.

A Localization of GFP-RBP and GFP-TCTP in root cells of 5-day-old *Arabidopsis* seedlings expressing *ProRBP:GFP-RBP* or *Pro35S:GFP-TCTP*. The seedlings were grown under 23°C (NS) or incubated at 39°C for 60 min (HS). Scale bars = 10 μm.

B Co-localization analysis of GFP-RBP (green) or GFP-TCTP (green) with eIF4E (red, α-eIF4E) in heat-stressed (39°C for 60 min) root tip cells of 5-day-old *Arabidopsis* seedlings expressing *ProUBP:UBP-GFP* or *Pro35S:TCTP-GFP*. Scale bars = 10 μm.

C FRET assay of the indicated protein combinations using CFP-YFP pair in *N. benthamiana* leaves under HS (39°C for 40 min). EV, empty vector (negative control). Upper and lower box boundaries represent the first and third quantiles, respectively. Horizontal lines mark the median of at least five replicate measurements, and whiskers mark the highest and lowest values. The experiment was repeated three times with similar results. *P* values denote statistically significant differences for comparisons to plants expressing EV (two-tailed *t*-test).

D Co-immunoprecipitation of RBP or TCTP with TSN in protein extracts prepared from 10-day-old *Arabidopsis* seedlings expressing *ProRBP:GFP-RBP* or *Pro35S:GFP-TCTP*. The GFP-expressing line was used as a negative control. Endogenous TSN (107 kD) was detected in the total fractions (Input) and in the fractions co-immunoprecipitated (Co-Ip) with RBP and TCTP but not with free GFP. Input and Co-Ip fractions were analysed by immunoblotting using α-TSN and α-GFP.

Source data are available online for this figure.

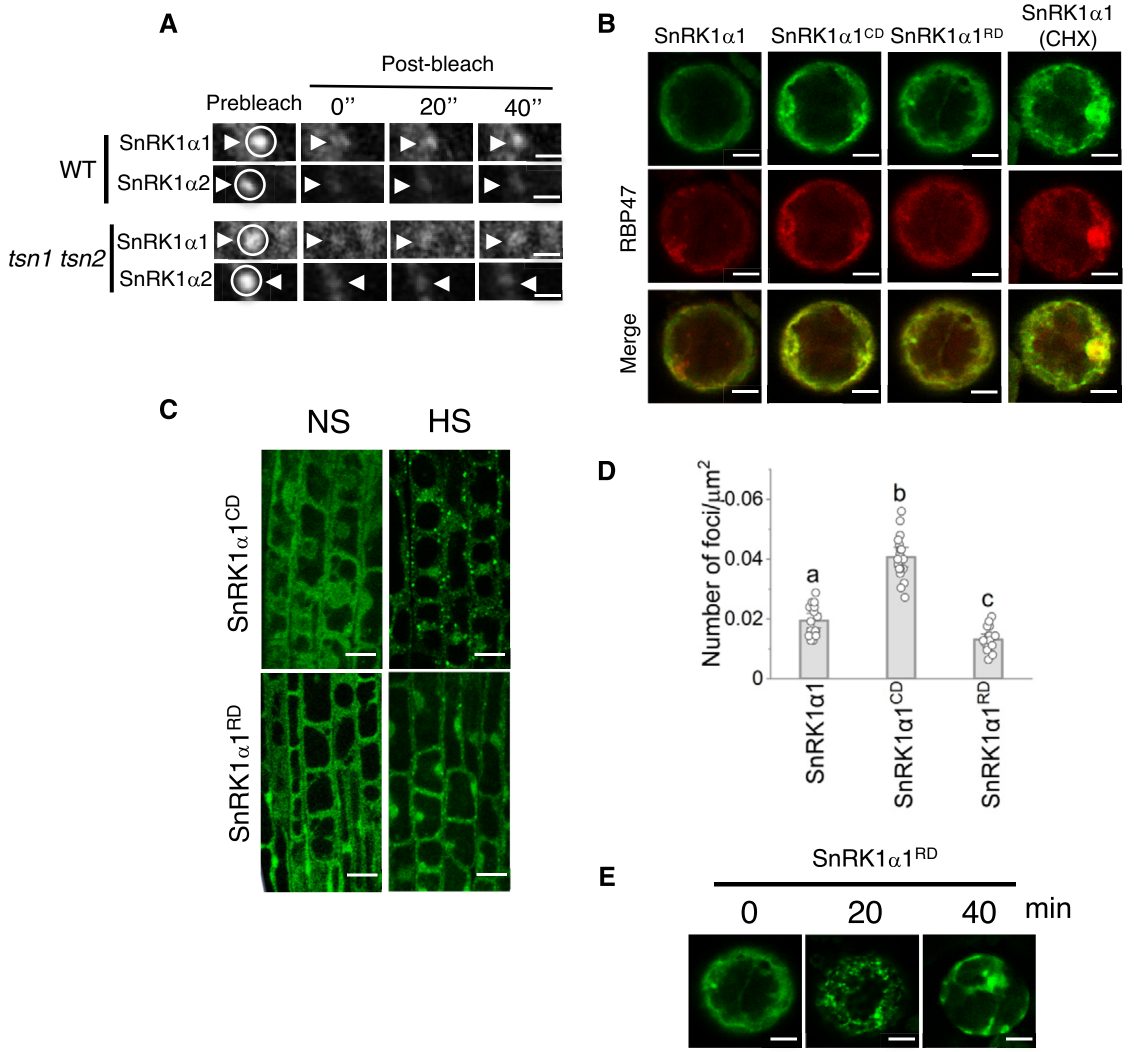

Figure EV5. Dynamics of SnRK1α in SGs.

A Selected time frames (0, 20 and 40 s after bleaching) from FRAP analysis of SnRK1α1 and SnRK1α2 in root tip cells of WT and *tsn1 tsn2* seedlings, both expressing either *ProUBQ:GFP-SnRK1α1* or *ProUBQ:GFP-SnRK1α2* after HS (39°C for 60 min). Circles and arrowheads indicate the bleached foci. The corresponding signal recovery rate ($t_{1/2}$) and proportion of the initial signal recovered (%) of GFP-tagged proteins are shown in Fig 7G and H, respectively. Scale bars = 3 µm.

B Co-localization of GFP-SnRK1α1 (with and without CHX treatment), GFP-SnRK1α1^{CD} or GFP-SnRK1α1RD with RFP-RBP47 in *N. benthamiana* protoplasts under NS conditions (23°C). Scale bars = 5 µm.

C Localization of GFP-SnRK1α1^{CD} and GFP-SnRK1α1RD in root tip cells of 5-day-old *Arabidopsis* WT seedlings expressing *ProUBQ:GFP-SnRK1α1^{CD}* and *ProUBQ:GFP-SnRK1α1RD* grown under 23°C (NS) or incubated at 39°C for 60 min (HS). Scale bars = 10 µm.

D Quantification of GFP foci in root tip cells of 5-day-old *Arabidopsis* WT seedlings expressing *ProUBQ:GFP-SnRK1α1*, *ProUBQ:GFP-SnRK1α1^{CD}* or *ProUBQ:GFP-SnRK1α1RD* after HS (39°C for 60 min). Data represent means ± SD of 15–25 measurements from three different experiments. Means with different letters are significantly different at $P < 0.001$ (one-way ANOVA).

E Kinetics of GFP-SnRK1α1RD in *N. benthamiana* protoplasts expressing *ProUBQ:GFP-SnRK1α1RD*. Protoplasts were kept under control conditions (0 min) or heat-stressed at 39°C for 20 and 40 min. Scale bars = 5 µm.

 © 2021 The Authors
