# Supplementary material for: Tudor staphylococcal nuclease is a docking platform for stress granule components and is essential for SnRK1 activation in Arabidopsis
Source: EMBO J. 2021 Jul 21;40(17):e105043. doi: 10.15252/embj.2020105043 (PMC8447601; doi:10.15252/embj.2020105043)
Supplement: Supplementary file 7 — Source Data for Expanded View and Appendix [file EMBJ-40-e105043-s010.zip › Appendix_and_EV_Figure_Source_data/FigureEV4_Source_Data.pdf]

Figure EV4D

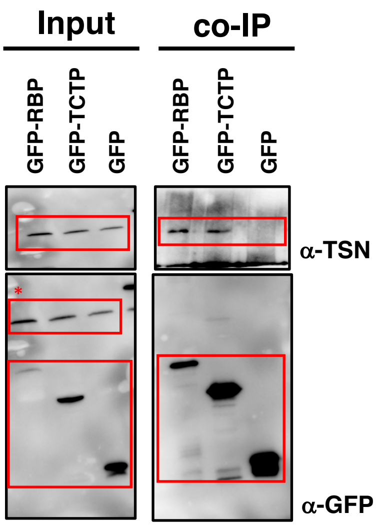

* The Membrane was incubated firstly with α-TSN and then with α-GFP, both from mouse
