# Supplementary figures and images for: Tudor staphylococcal nuclease is a docking platform for stress granule components and is essential for SnRK1 activation in Arabidopsis
Source: EMBO J. 2021 Jul 21;40(17):e105043. doi: 10.15252/embj.2020105043 (PMC8447601; doi:10.15252/embj.2020105043)

Appendix Figure S7B

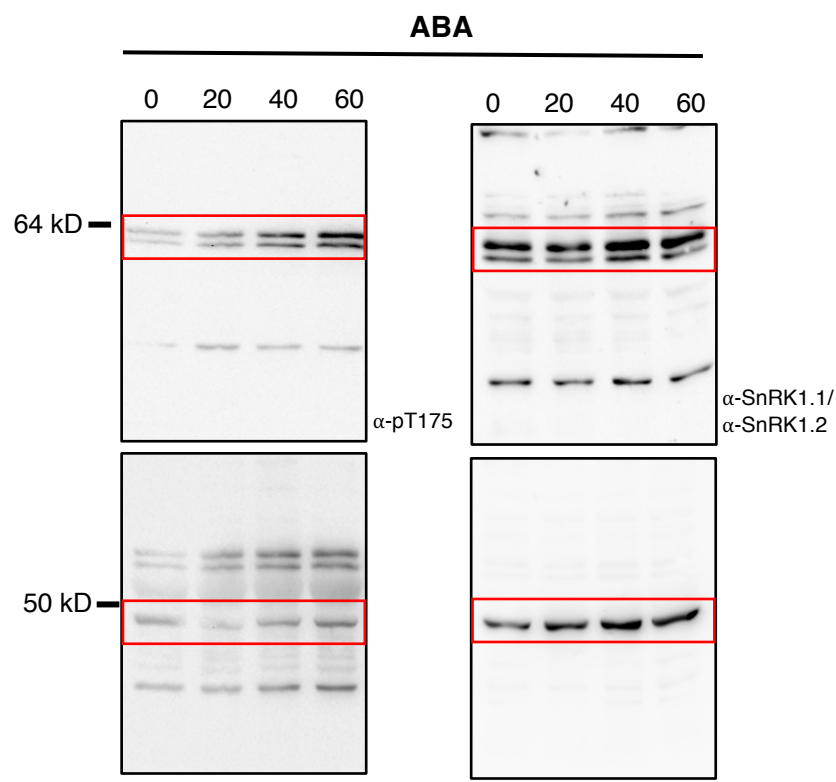

Supplement: Supplementary file 7 — Source Data for Expanded View and Appendix [file EMBJ-40-e105043-s010.zip › Appendix_and_EV_Figure_Source_data/Appendix_FigS7_Source_Data.pdf]

Figure EV1B

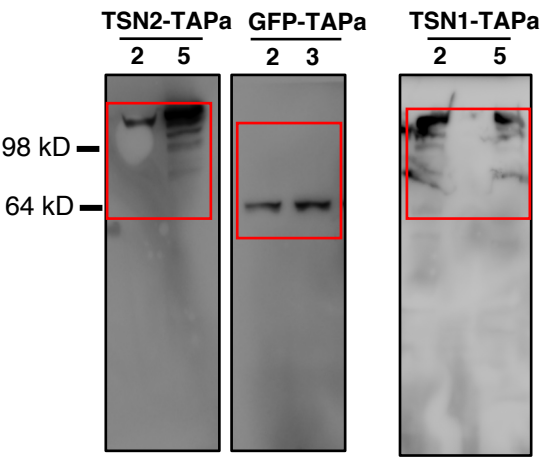

Figure EV1E

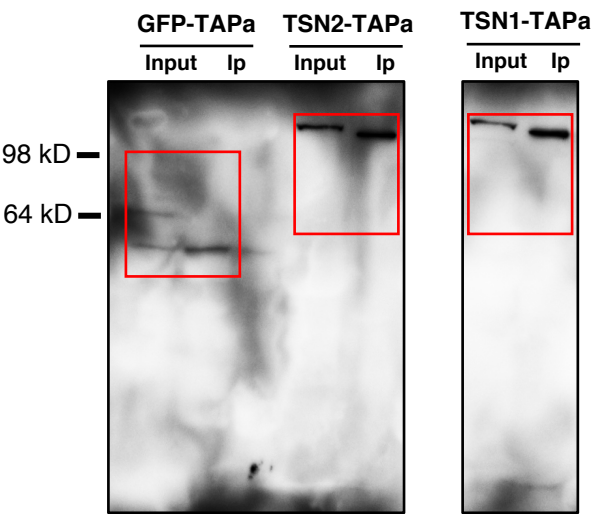

Supplement: Supplementary file 7 — Source Data for Expanded View and Appendix [file EMBJ-40-e105043-s010.zip › Appendix_and_EV_Figure_Source_data/FigureEV1_Source_Data.pdf]

Figure 5B

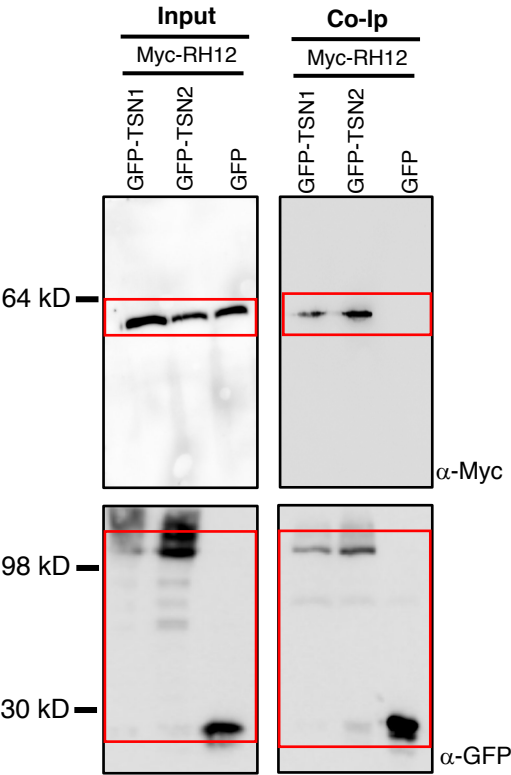

Supplement: Supplementary file 8 — Source Data for Figure 5B [file EMBJ-40-e105043-s004.pdf]

Figure 6B

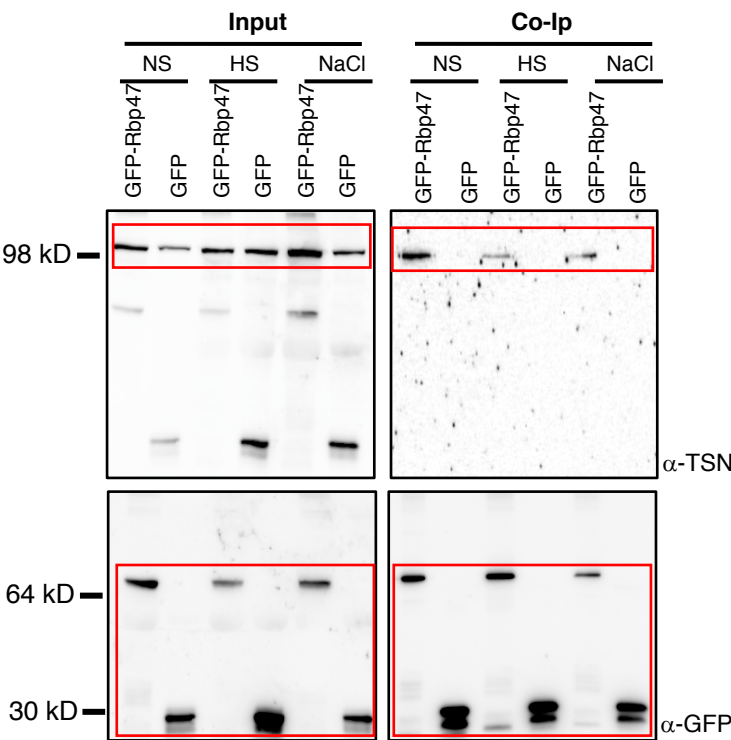

Supplement: Supplementary file 9 — Source Data for Figure 6B [file EMBJ-40-e105043-s007.pdf]

Figure 7A

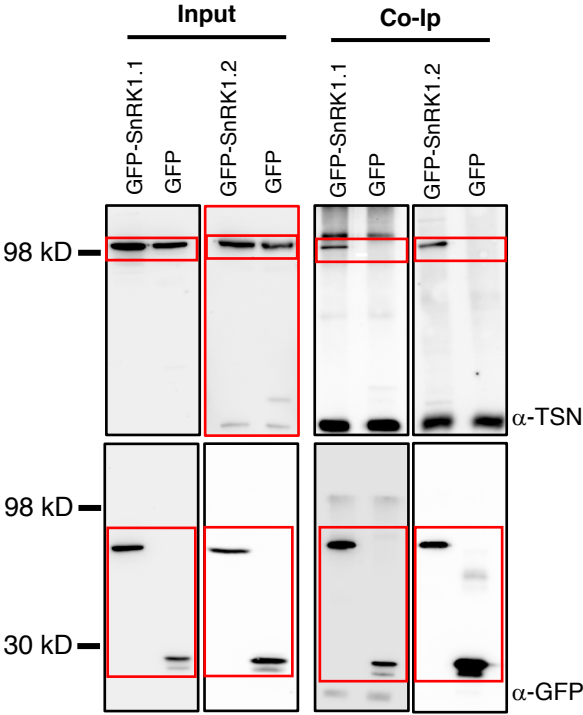

Figure 7B

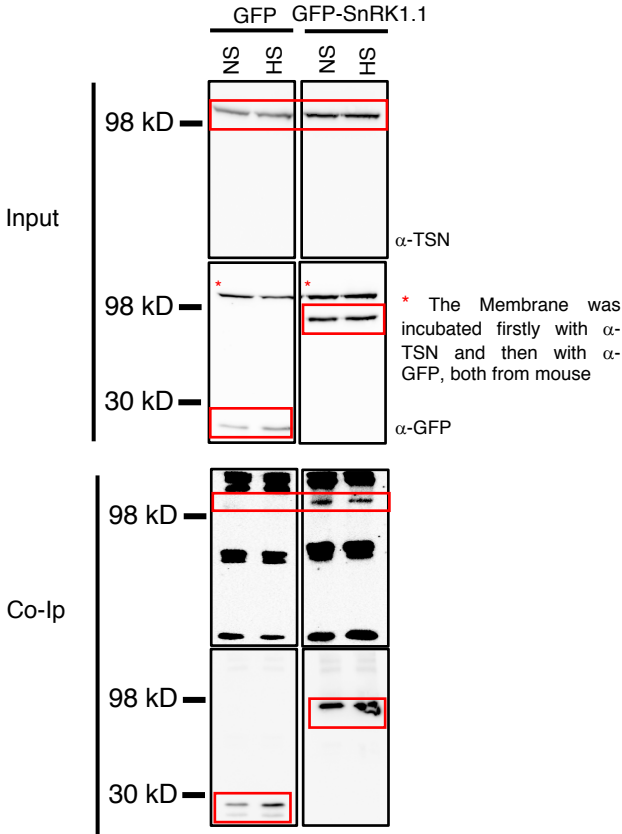

Supplement: Supplementary file 10 — Source Data for Figure 7A,B [file EMBJ-40-e105043-s005.pdf]

Figure 8A

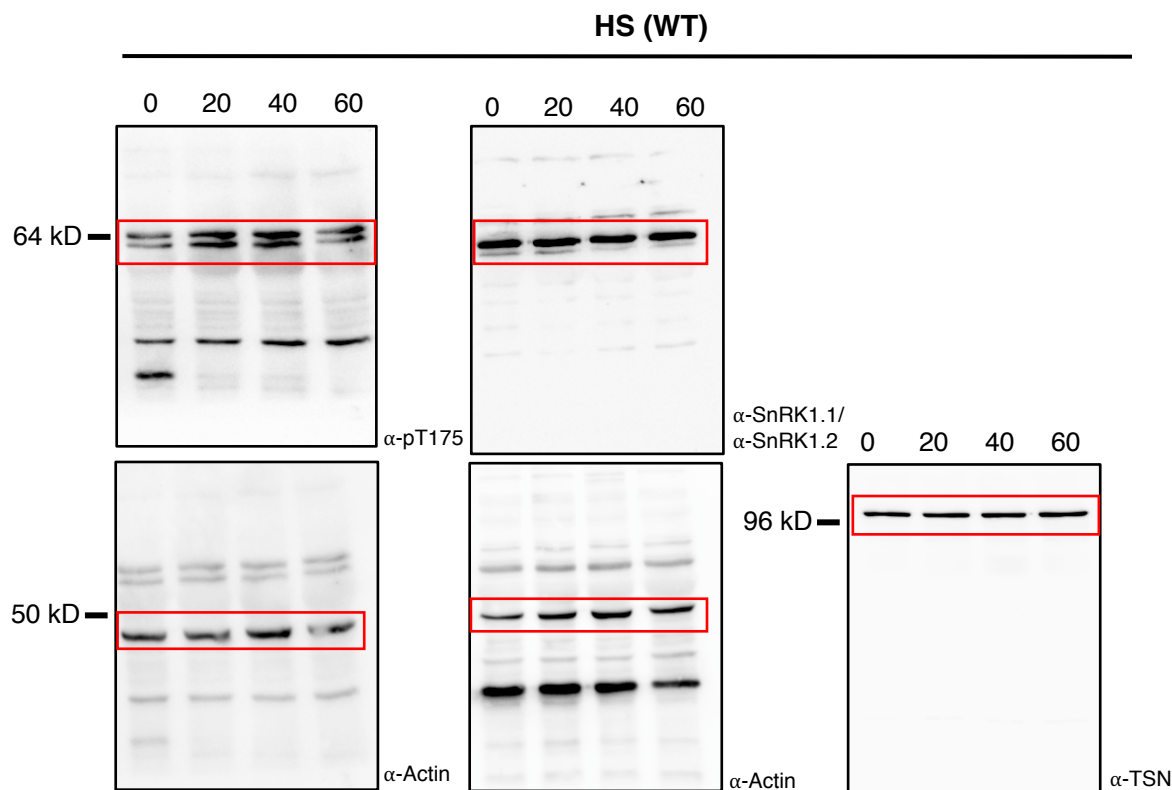

Figure 8B

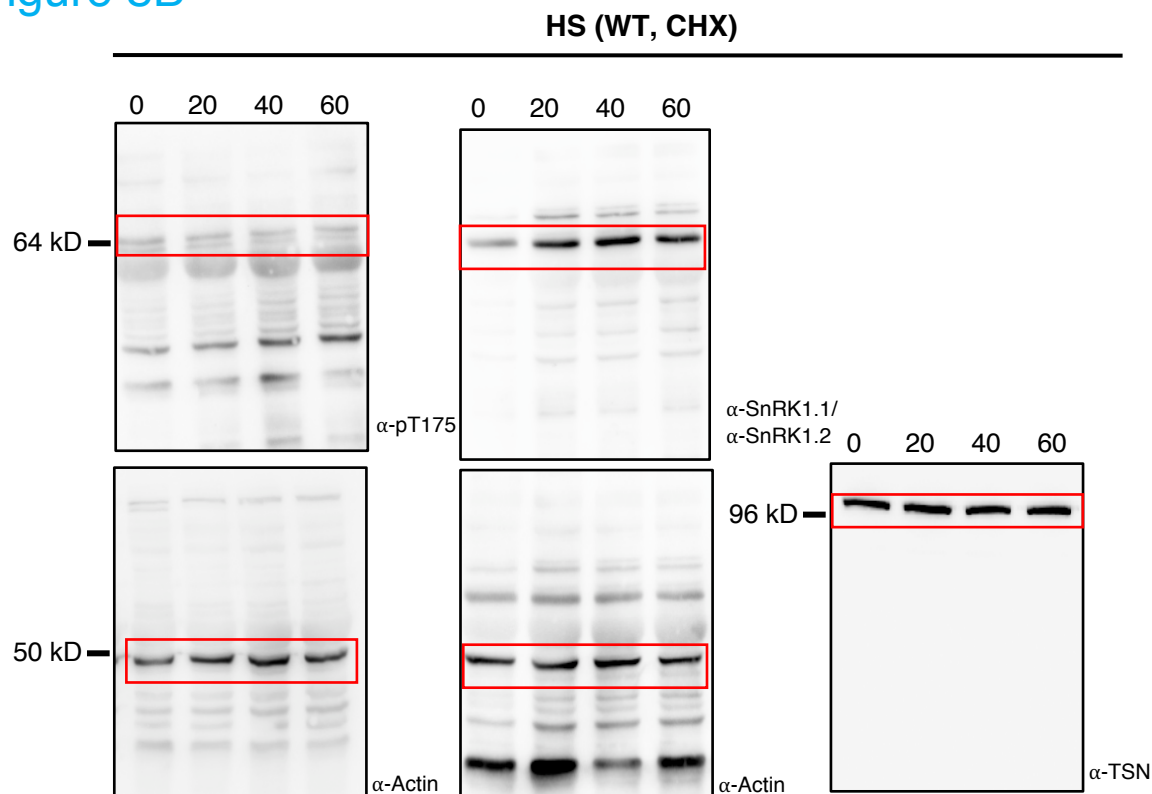

Figure 8E

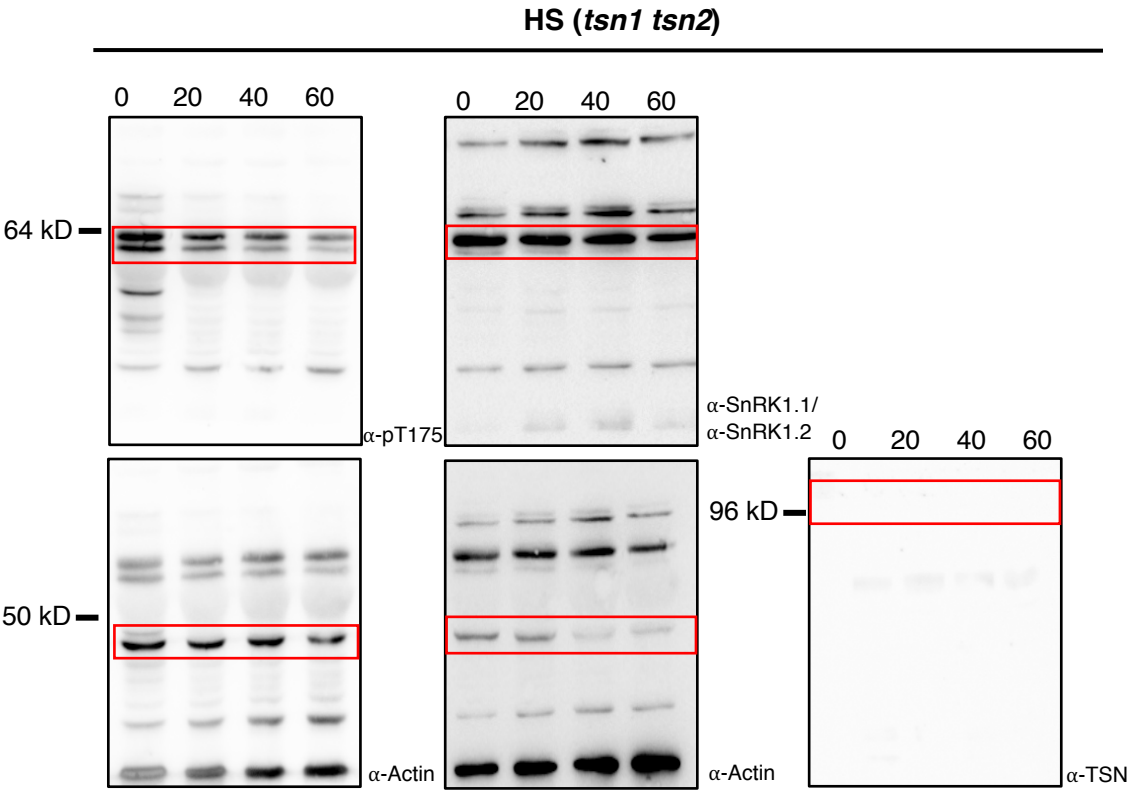

Figure 8F

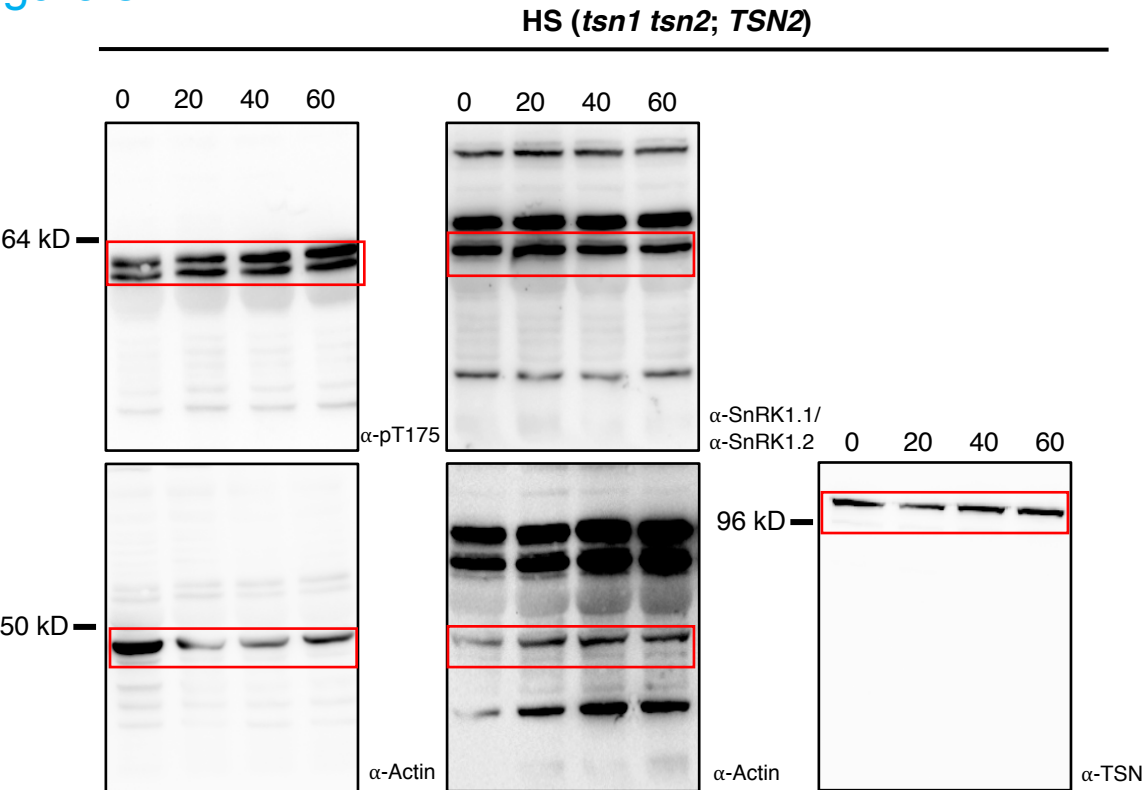

Supplement: Supplementary file 11 — Source Data for Figure 8 [file EMBJ-40-e105043-s008.pdf]
